# The Multifaceted Role of LRRK2 in Parkinson’s Disease

**DOI:** 10.3390/brainsci15040407

**Published:** 2025-04-17

**Authors:** Dong Hwan Ho, Sun Jung Han, Ilhong Son

**Affiliations:** 1 InAm Neuroscience Research Center, Wonkwang University Sanbon Medical Center, 321, Sanbon-ro, Gunpo-si 15865, Gyeonggi-do, Republic of Korea; ethan2887@gmail.com; 2 Department of Neurology, College of Medicine, Wonkwang University, 321, Sanbon-ro, Gunpo-si 15865, Gyeonggi-do, Republic of Korea; sunjung516@hanmail.net

**Keywords:** Parkinson’s disease, leucine-rich repeat kinase 2, mitochondrial homeostasis, translation, cellular senescence, neuroinflammation, ciliogenesis, neurotrophic factor

## Abstract

Leucine-rich repeat kinase 2 (LRRK2) is a multifunctional protein kinase intricately involved in the pathogeneses of various neurodegenerative diseases, particularly Parkinson’s disease (PD). LRRK2 plays a pivotal role in mitochondrial function and cellular senescence by regulating key processes such as autophagy, oxidative stress, and protein aggregation. LRRK2 is also associated with ciliogenesis in regulating neuronal development. In addition, LRRK2 has been implicated as a putative mediator in neuroinflammation via promoting the reactivation of microglia and influencing cytokine production, a factor that may have therapeutic implications. Furthermore, mutations in LRRK2 have been found to impact the production of neurotrophic factors in astrocytes, the star-shaped glial cells of the central nervous system, thereby affecting neuronal health and contributing to the pathology of neurodegenerative diseases like PD. The multifaceted roles of LRRK2 in cellular senescence, interaction with LRS, neuroinflammation, the maintenance of mitochondria, and astrocyte function highlight its significance as a therapeutic target for neurodegenerative disorders.

## 1. Introduction

Parkinson’s disease (PD) is a progressive neurological disorder affecting movement control. It occurs due to the degeneration of dopamine-producing neurons in the substantia nigra, a brain region essential for regulating movement [1]. This dopamine deficiency leads to symptoms such as tremors, bradykinesia, rigidity, and postural instability. Beyond motor symptoms, patients often experience non-motor issues like depression, anxiety, sleep disturbances, and cognitive decline [2]. The exact cause of PD is not well understood but is believed to involve a combination of genetic and environmental factors. Some genetic mutations increase the risk, while exposure to toxins and head injuries may also contribute to it. The condition typically manifests in individuals over the age of 60, though early-onset cases can occur [3]. There is currently no cure for PD, but there are treatments designed to manage symptoms. Medications such as L-DOPA, MAO-B inhibitors, and dopamine agonists are commonly prescribed. Physical therapy and occupational therapy can help improve mobility and daily functioning. In some cases, surgical interventions like deep brain stimulation may be considered [4]. Ongoing research continues to explore new treatments, including stem cell therapy and neuroprotective agents, with the hope of slowing or halting the disease’s progression [5].

**Figure 1:**
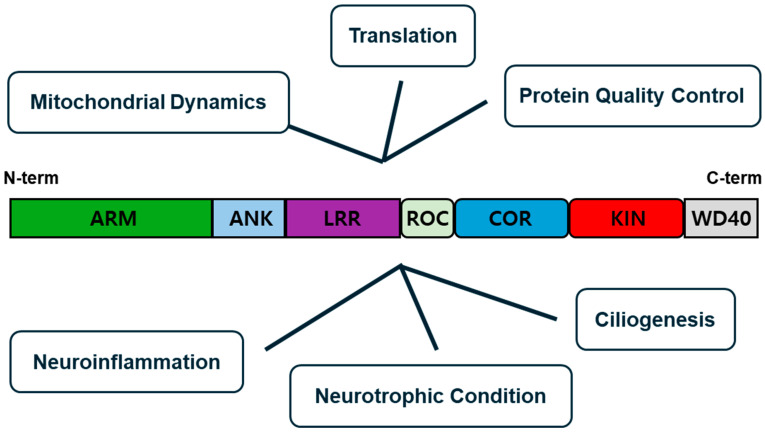
The structure of LRRK2 and its cellular function. Leucine-rich repeat kinase 2 (LRRK2) protein is composed of armadillo repeat (ARM), ankyrin repeat (ANK), leucine-rich repeat (LRR), Ras-of-complex (ROC), c-terminal of ROC (COR), kinase (KIN), and WD40. LRRK2 has been implicated in cellular mechanisms such as mitochondrial dynamics, translation, protein quality control, neuroinflammation, astrocytic neurotrophic conditioning, and ciliogenesis.

Leucine-rich repeat kinase 2 (LRRK2) is a gene that plays a crucial role in various cellular processes by encoding a large and multifunctional enzyme (Figure 1). Identified in the early 2000s, LRRK2 came into prominence when researchers linked mutations in the gene to PD [6]. LRRK2 is expressed in various brain cells, including neurons (especially in dopamine-rich areas like the putamen and substantia nigra) and glial cells (astrocytes, microglia, and oligodendroglia). Its levels and specific functions can vary by cell type and brain region. The most prevalent mutation associated with PD is the G2019S mutation, which is found in both familial and sporadic cases. LRRK2 encodes a protein that contains several functional domains, including a kinase domain (KIN) that adds phosphate groups to other proteins and a GTPase domain (ROC and COR) that hydrolyzes GTP, a molecule involved in energy transfer within cells [7]. These domains suggest that LRRK2 is involved in a multitude of signaling pathways that regulate cellular functions such as mitochondrial function, vesicular transport, cellular senescence, autophagy, and immune response [8]. Herein, we aim to elucidate the intracellular functions of LRRK2 and discuss the various LRRK2-mediated PD pathomechanisms.

## 2. The Association Between LRRK2 and Mitochondria

### 2.1. LRRK2-Involved Mitochondrial Homeostasis and Dynamics

Mitochondria are essential organelles responsible for ATP production, calcium homeostasis, and apoptotic regulation [9]. LRRK2 plays a pivotal role in maintaining mitochondrial homeostasis, a balance critical for cellular health [10]. LRRK2 is localized to mitochondria-associated membranes, facilitating interactions between mitochondria and other cellular organelles such as the endoplasmic reticulum (ER) [11]. These interactions are vital for calcium exchange, lipid synthesis, and cellular signaling. Mutations in LRRK2, such as the G2019S and R1441C mutations, have been shown to disrupt mitochondrial functions, leading to bioenergetic deficits and increased oxidative stress [12,13,14]. Studies have demonstrated that LRRK2’s kinase activity is crucial for modulating mitochondrial processes, including oxidative phosphorylation and ATP production. Dysregulation of these processes due to LRRK2 mutations results in compromised mitochondrial function and contributes to neuronal degeneration in PD [6,15,16].

**Figure 2:**
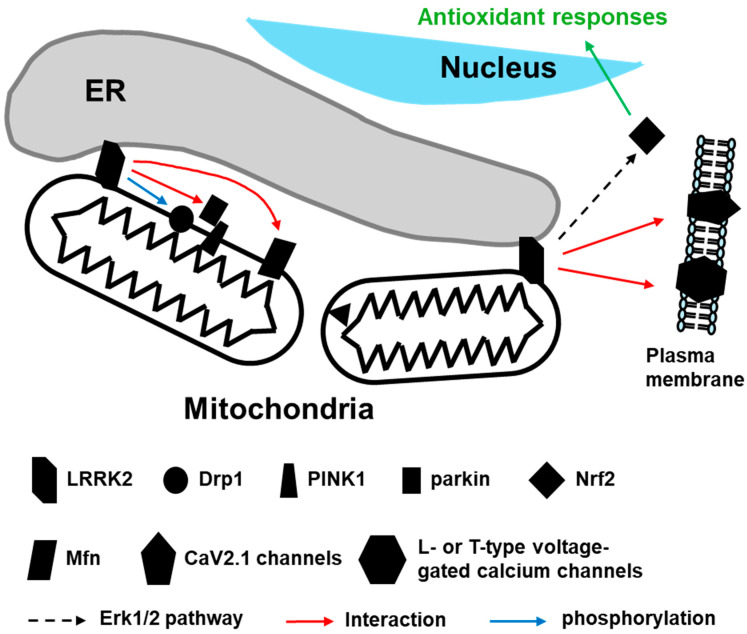
The involvement of LRRK2 in mitochondrial dynamics and homeostasis. LRRK2-phosphorylated dynamin-related protein 1 (Drp1) and the interaction of LRRK2 with mitofusins (Mfn) or Parkin, the latter of which binds to PTEN-induced kinase 1 (PINK1), are related to mitochondrial dynamics. The LRRK2-mediated Erk1/2 pathway is linked to the activation of nuclear factor-like 2 (Nrf2), contributing to anti-oxidative stress responses and mitochondrial calcium transport. LRRK2 has also been demonstrated to be associated with the regulation of calcium channels in the plasma membrane.

Mitochondrial dynamics, encompassing fission, fusion, and motility, are essential for maintaining mitochondrial integrity and function. LRRK2 has been found to regulate mitochondrial dynamics by interacting with key proteins involved in these processes. One such protein is dynamin-related protein 1 (Drp1), a critical regulator of mitochondrial fission. LRRK2 phosphorylates Drp1, modulating its activity and influencing mitochondrial fission (Figure 2) [17,18]. Mutations in LRRK2 have been shown to alter Drp1 activity, leading to aberrant mitochondrial fragmentation [10,19]. This abnormal mitochondrial morphology is a hallmark of PD and believed to contribute to neuronal degeneration.

In addition to its role in fission, LRRK2 influences mitochondrial fusion through interactions with proteins like Mitofusin (Mfn) [20,21,22]. These proteins are essential for the fusion of the outer and inner mitochondrial membranes (Figure 2). Dysregulation of mitochondrial fusion due to LRRK2 mutations results in fragmented and dysfunctional mitochondria, exacerbating cellular stress and neuronal damage.

Mitophagy, the selective autophagic clearance of damaged mitochondria, is a critical process for maintaining mitochondrial quality and cellular health. The interplay between LRRK2 and Parkin plays a vital role in neuronal degeneration (Figure 2) [23]. Under normal conditions, PTEN-induced kinase 1 (PINK1) accumulates on the outer mitochondrial membrane of damaged mitochondria, recruiting Parkin to initiate mitophagy. Mutations in LRRK2 disrupt this process, resulting in the accumulation of dysfunctional mitochondria [24,25]. The impaired clearance of damaged mitochondria exacerbates cellular stress and contributes to neurodegeneration in PD. The interplay between LRRK2 and mitophagy highlights the importance of mitochondrial quality control in neuronal health and disease.

### 2.2. LRRK2 in Mitochondrial Ca^2+^ Handling and Oxidative Stress

Proper mitochondrial calcium ion (Ca^2+^) handling is crucial for cellular signaling, bioenergetics, and apoptosis regulation [26]. LRRK2 mutations have been implicated in dysregulated mitochondrial Ca^2+^ responses, leading to impaired energy metabolism and increased susceptibility to cellular stress (Figure 2). Dysregulated mitochondrial Ca^2+^ handling due to LRRK2 mutations can lead to elevated intracellular Ca^2+^ levels, triggering a cascade of detrimental effects, including mitochondrial dysfunction, oxidative stress, and apoptosis. These effects are particularly pronounced in dopamine neurons, which are highly vulnerable to Ca^2+^ dysregulation [14]. Studies have shown that LRRK2 interacts with key calcium-handling proteins, modulating the activity of CaV2.1 channel or L- or T-type voltage-gated calcium channels and influencing mitochondrial Ca^2+^ uptake and release via the ERK1/2 pathway [27,28,29]. The interplay between LRRK2 and mitochondrial Ca^2+^ handling underscores this kinase’s role in maintaining neuronal health and its contribution to the pathogenesis of PD.

Oxidative stress results from an imbalance between reactive oxygen species (ROS) production and antioxidant defenses, and elevated ROS levels can damage cellular components [30]. LRRK2 activity is linked to oxidative stress, as it influences mitochondrial function and the generation of ROS, and mutant LRRK2 variants are associated with increased oxidative damage [15,31]. Mitochondria constitute the primary source of ROS within cells [32]. LRRK2 has been shown to affect mitochondrial dynamics and integrity, and dysregulation of these processes via LRRK2 mutations leads to mitochondrial dysfunction and increased ROS production [33,34]. Cells possess antioxidant defense mechanisms to counteract ROS. LRRK2 has been implicated in the regulation of these defenses, particularly through the nuclear factor-like 2 (Nrf2) pathway (Figure 2) [35]. Nrf2 is a transcription factor that activates the expression of antioxidant genes. LRRK2’s phosphorylation of Nrf2 and its inhibitors influences Nrf2 activity and cellular antioxidant capacity [36]. Mutant LRRK2 variants impair Nrf2 signaling, reducing antioxidant defenses and exacerbating oxidative stress [37].

Mitochondria are vital for energy production and cellular health, and LRRK2 is key in maintaining mitochondrial balance. LRRK2 mutations disrupt mitochondrial functions, causing energy deficits and oxidative stress, linked to PD. Furthermore, LRRK2 regulates mitochondrial dynamics and mitophagy, helping clear damaged mitochondria.

## 3. The Interplay Between LRRK2 and Translation

### 3.1. Regulation of mRNA Translation by LRRK2

**Figure 3:**
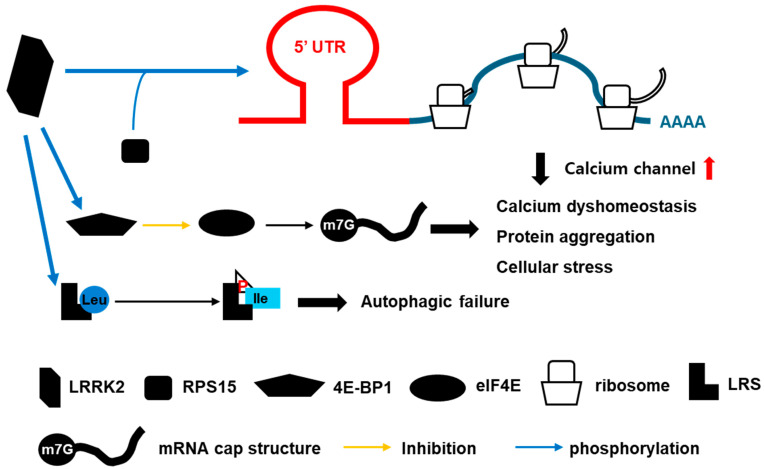
The role of LRRK2 in translation. LRRK2 phosphorylates ribosomal protein S15 (RPS15), contributing to the mRNA translation process, and facilitates the translation of the 5′ untranslated region (5′ UTR), resulting in the upregulation of calcium channel expression. Furthermore, the phosphorylation of 4E-BP1 by LRRK2 enhances the eIF4E-mediated initiation of mRNA translation, thereby increasing protein aggregation and cellular stress. LRRK2 has also been demonstrated to promote autophagic failure via the phosphorylation of leucyl-tRNA synthetase (LRS). “P” with red letter in the triangle is the phosphorylation state of LRRK2.

LRRK2’s regulation of mRNA translation is crucial, especially in neurons. The G2019S mutation, which is often associated with familial PD, has been shown to enhance the translation of mRNAs via ribosomal protein S15 (RPS15) in both cap-dependent and cap-independent mRNA translation [38]. Translational dysregulation caused by LRRK2 mutations can be attributed to several mechanisms. One key mechanism involves the activation of translation initiation factors. LRRK2 interacts with and phosphorylates 4E-BP1, a binding inhibitor of the eukaryotic initiation factor 4E (eIF4E), enhancing the binding of eIF4E to the cap structures of mRNAs and promoting translation initiation. G2019S mutations in LRRK2 can lead to hyperphosphorylation, resulting in increased translation of specific mRNAs [39]. Additionally, LRRK2 mutations can influence mRNAs’ stability, particularly those with complex 5′ untranslated region (5′ UTR) structures. Increased mRNA stability leads to prolonged translation and increased protein synthesis, contributing to the accumulation of proteins that disrupt cellular homeostasis and promote neurodegeneration (Figure 3). Ribosome profiling studies have demonstrated that the G2019S LRRK2 mutation significantly alters the translation of numerous mRNAs, suggesting that LRRK2 has a broad impact on the translational landscape [40]. These alterations indicate that LRRK2 mutations can lead to widespread translational defects, contributing to cellular dysfunction and neurodegeneration in PD. These mRNAs often encode proteins essential for calcium homeostasis and stress responses. A significant consequence of dysregulated translation driven by LRRK2 mutations is calcium dysregulation in dopamine neurons. Calcium homeostasis is critical for neuronal health and function, and disruptions in calcium signaling are a hallmark of PD. Increased translation of mRNAs encoding calcium channels and related proteins leads to elevated intracellular calcium levels (Figure 3). Studies using LRRK2 knock-out (KO) mouse models have further elucidated LRRK2’s role in translation. The absence of LRRK2 leads to the downregulation of mRNAs with complex 5′UTR structures, highlighting this kinase’s role in promoting the translation of these specific mRNAs [41]. These findings underscore the importance of LRRK2’s kinase activity in regulating mRNA translation and maintaining cellular homeostasis.

### 3.2. Leucyl-tRNA Synthetase (LRS) and Its Interaction with LRRK2

The primary function of leucyl-tRNA synthetase (LRS) is to catalyze the attachment of leucine to tRNA-Leu, forming leucyl-tRNA, which is essential for ribosomal translation. This process ensures that leucine is accurately incorporated into proteins, maintaining the fidelity of protein synthesis [42]. Beyond its role in protein synthesis, LRS functions as a leucine sensor in the mammalian target of rapamycin complex 1 (mTORC1) pathway. mTORC1 is a critical regulator of cell growth, metabolism, and autophagy. By sensing leucine availability, LRS activates mTORC1, which, in turn, regulates various cellular processes [43]. This function positions LRS as a key player in nutrient sensing and metabolic regulation. LRS is involved in the regulation of autophagy, a cellular process that degrades and recycles damaged proteins and organelles. By activating mTORC1, LRS can inhibit autophagy under nutrient-rich conditions. Conversely, the downregulation of LRS can induce autophagy, helping to maintain cellular homeostasis [44,45]. This balance is crucial for cell survival and function, particularly under stress conditions. LRS plays a role in myogenic differentiation and skeletal muscle regeneration. It negatively regulates myoblast differentiation, and its downregulation accelerates muscle regeneration in injury models [46]. This non-translational function of LRS is independent of its role in protein synthesis and involves the Rag-mTORC1 pathway. The ability of LRS to influence muscle biology highlights its versatility beyond protein synthesis. LRRK2 can phosphorylate LRS at threonine 293 (Thr293) in its editing domain. This phosphorylation impairs LRS’s ability to edit mischarged tRNAs, leading to increased protein misfolding and ER stress [47]. The G2019S mutation in LRRK2, which enhances its kinase activity, exacerbates this effect. The phosphorylation of LRS by LRRK2 disrupts its normal function, resulting in impaired autophagy. This leads to the accumulation of α-synuclein and its aggregates, which are hallmarks of neurodegenerative diseases like PD [47]. Phosphorylated LRS exhibits defective leucine binding and editing functions, contributing to ER stress and the accumulation of autophagy markers such as LC3B-II and p62. This stress response is further evidenced by increased levels of GRP78/BiP, a chaperone protein involved in protein folding [47]. The chronic ER stress and protein aggregation associated with LRS phosphorylation by LRRK2 underscore the significance of this interaction in PD pathogenesis. The interaction between LRRK2 and LRS highlights the role of LRRK2 in modulating autophagy and protein homeostasis through LRS (Figure 3).

LRRK2 plays a vital role in regulating mRNA translation, especially in neurons. The G2019S mutation enhances mRNA translation via ribosomal protein S15, affecting translation initiation and mRNA stability. This can lead to calcium dysregulation and contributes to cellular dysfunction in PD. And the phosphorylation of LRS by LRRK2, resulting in LRS malfunctions, leads to increases in misfolded protein quantities and ER stress.

## 4. The Role of LRRK2 in Protein Quality Control

### 4.1. Autophagy Regulation

**Figure 4:**
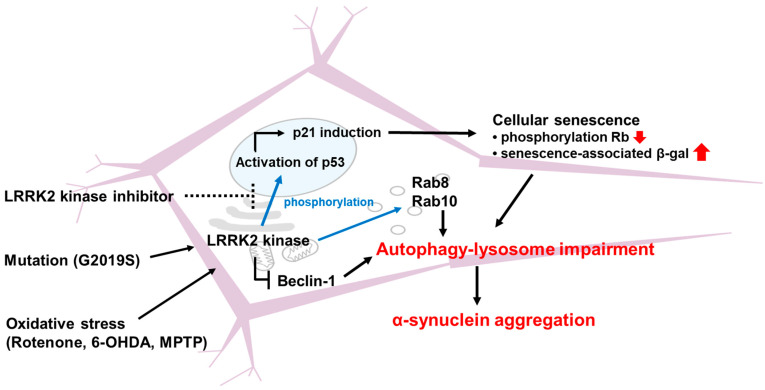
The function of LRRK2 in protein quality control. LRRK2 has been demonstrated to impede the autophagy–lysosomal pathway by suppressing Beclin-1 and augmenting the phosphorylation of Rab GTPases, including Rab8, Rab 10, and Rab35. The elevation of LRRK2 kinase activity by specific mutations like G2019S and oxidative stress, such as that generated by rotenone, 6-hydroxydopamine, and 1-methyl-4-phenyl-1,2,3,6-tetrahydropyridine (MPTP), have been shown to highly phosphorylate p53. Activation of p53 induces the activity of p21, and p53–p21 cell-cycle arrest axis drives cellular senescence via decreasing Rb phosphorylation and increasing senescence-associated beta-galactosidase (senescence-associated β-gal) in dopaminergic neurons, thereby aggravating autophagy–lysosomal impairment and α–synuclein aggregation.

Autophagy is a cellular process that degrades and recycles damaged organelles and proteins, maintaining cellular homeostasis [48]. Studies suggest that LRRK2 mutations impair autophagic flux, leading to a build-up of toxic protein aggregates and cellular stress. LRRK2 has been shown to regulate autophagy through interaction with Beclin-1, which is essential for the formation of autophagosomes [49]. LRRK2’s phosphorylation of Rab GTPases modulates their activity, influencing autophagy initiation and progression [50,51,52]. Furthermore, LRRK2 interacts with lysosomal proteins, affecting autophagosome-lysosome fusion and cargo degradation [53,54]. Mutations in LRRK2, such as the G2019S and R1441C variants, have been shown to disrupt autophagic processes [55,56]. These mutations enhance LRRK2 kinase activity, leading to hyperphosphorylation of autophagy-related proteins and impairing their function. Consequently, mutant LRRK2-expressing cells exhibit reduced autophagic flux, resulting in the accumulation of damaged organelles and protein aggregates (Figure 4). This disruption contributes to cellular stress and senescence, particularly in neurons.

### 4.2. Aggregation of α-Synuclein

α-Synuclein is a presynaptic protein that can misfold and aggregate under pathological conditions, and its aggregates form Lewy bodies, which are considered a hallmark of PD [57]. LRRK2 regulates the degradation of α-synuclein through the autophagy–lysosome pathway. Cellular senescence caused by LRRK2 kinase activation is characterized by a lack of α-synuclein degradation because lysosomal activity is impaired by cellular senescence. And G2019S mutant LRRK2 impairs α-synuclein degradation, leading to the accumulation of α-synuclein aggregates via aberrant Rab regulation and cellular senescence (Figure 4) [58,59]. Taken together, the vicious cycle involving the interplay of cellular senescence triggered by LRRK2 kinase activation and the subsequent α-synuclein aggregation resulting from cellular senescence exacerbates the degeneration of dopaminergic neurons.

### 4.3. Cellular Senescence Caused by LRRK2

Cellular senescence is a state of irreversible cell cycle arrest characterized by changes in gene expression, morphology, and metabolic activity [60]. It contributes to aging and the development of age-related diseases, including neurodegenerative disorders [61]. Senescent cells exhibit distinct biomarkers, including the expression of p53, p21, and senescence-associated beta-galactosidase (SA-β-gal) [62]. LRRK2 activity influences the regulation of these markers. LRRK2 phosphorylates p53 in neurons, and increased LRRK2 kinase activity promotes the activation of p53 and upregulates p21 expression, promoting the senescence phenotype [63]. Additionally, SA-β-gal activity is elevated in cells expressing mutant LRRK2, further supporting its role in senescence [59]. The p53-p21 pathway is a key regulator of cellular senescence. p53 is a tumor suppressor protein that responds to cellular stress by inducing cell cycle arrest or apoptosis. p21, a cyclin-dependent kinase inhibitor, is a downstream target of p53 and mediates cell cycle arrest [64]. Mutant LRRK2 variants enhance p53 activation, leading to increased p21 expression and cellular senescence [59]. SA-β-gal is a widely used marker for cellular senescence. It is a lysosomal enzyme that accumulates in senescent cells, reflecting increased lysosomal activity and cellular aging. Studies have shown that LRRK2 activity influences SA-β-gal expression. Mutant LRRK2 variants increase lysosomal biogenesis and SA-β-gal activity, promoting the senescence phenotype [58]. The role of LRRK2 in lysosomal function further underscores its involvement in cellular senescence (Figure 4).

LRRK2 plays a crucial role in maintaining protein quality control, specifically through regulating autophagy. Mutations in LRRK2 can impair autophagic processes and promote toxic protein accumulation, leading to cellular stress, especially in neurons. LRRK2 also influences the degradation of α-synuclein, and its activation is linked to cellular senescence through the phosphorylation of p53 and induction of p21.

## 5. The Role of LRRK2 in Neuroinflammation

### 5.1. LRRK2 and the Activation of Microglia

**Figure 5:**
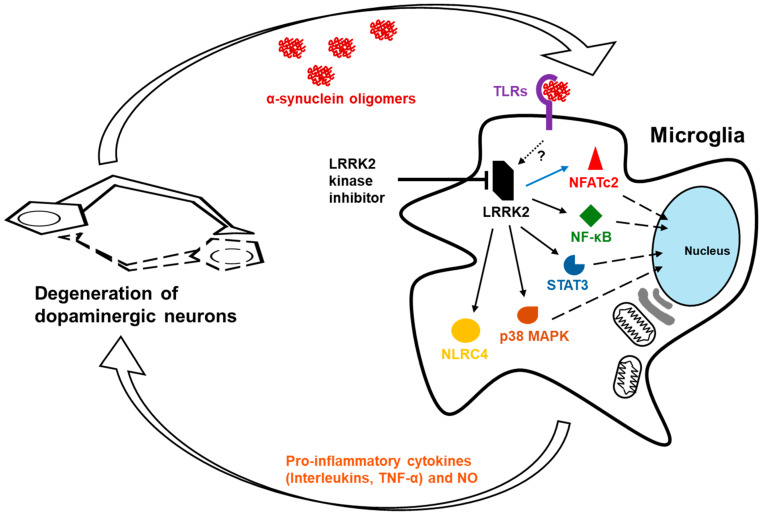
The action of LRRK in neuroinflammation. LRRK2 enhances the expression of proinflammatory cytokines, including tumor necrosis factor-alpha (TNF-α) and interleukins, and nitric oxide (NO) via interactions with the transcription factors nuclear factor kappa-light-chain-enhancer of activated B cells (NF-κB), p38 mitogen-activated protein kinase (p38 MAPK), and signal transducer and activator of transcription 3 (STAT3) in microglia. LRRK2-mediated phosphorylation of nuclear factor of activated T cells 2 (NFATc2), which is driven by the stimulation of toll-like receptors (TLRs) with neuron-released α-synuclein, has been shown to increase proinflammatory responses. LRRK2 promotes the release of interleukins via regulating NLR family CARD domain-containing protein 4 (NLRC4). These neuroinflammatory responses are responsible for the degeneration of dopaminergic neurons. Phosphorylation by LRRK2 is indicated by the blue line.

Neuroinflammation is a complex, multifaceted response of the central nervous system (CNS) to injury, infection, or disease. It involves the activation of glial cells, the release of inflammatory mediators, and the recruitment of immune cells to the site of damage [65]. While acute neuroinflammation can be protective, chronic neuroinflammation is associated with neuronal damage and the progression of neurodegenerative diseases [66]. Microglia are the primary immune cells in the CNS, responsible for monitoring and maintaining homeostasis [67]. They become activated in response to injury or disease, adopting either a pro-inflammatory (M1) or anti-inflammatory (M2) phenotype [68]. LRRK2 is highly expressed in microglia, and its activity has been linked to microglial activation [69]. LRRK2 can influence microglial activation through several signaling pathways, which are critical for producing pro-inflammatory cytokines [70]. LRRK2’s phosphorylation of downstream targets modulates their activity, influencing the balance between pro-inflammatory and anti-inflammatory responses (Figure 5).

### 5.2. Cytokine Production

Activated microglia release a variety of cytokines and chemokines, which can exert a dual effect on neuronal health, one both protective and detrimental. Pro-inflammatory cytokines, including tumor necrosis factor-alpha (TNF-α), interleukin-1beta (IL-1β), IL-6, and IL-8, have been observed to exacerbate neuronal damage and contribute to the progression of neurodegenerative diseases [71]. LRRK2 activity has been demonstrated to influence the production of cytokines by microglia. Research suggests that LRRK2 mutations, such as the G2019S variant, can enhance the activation of p38 mitogen-activated protein kinase (MAPK). The activation of p38 MAPK contributes to neuroinflammation and neuronal damage [72]. Experiments involving LRRK2-knockdown microglia (brain immune cells) have shown reduced inflammatory responses. For instance, LRRK2 knockdown has been shown to result in a diminished production of inflammatory markers such as TNF-α and IL-1β as well as reduced p38 MAPK activation [72]. LRRK2 has been implicated in enhancing nuclear factor kappa-light-chain-enhancer of activated B cells (NF-κB) activity, which in turn promotes the production of pro-inflammatory cytokines such as IL-1β and IL-8. This suggests that LRRK2 may act as a modulator of NF-κB-mediated inflammatory signaling [73,74]. LRRK2 has been shown to phosphorylate nuclear factor of activated T-cells cytoplasmic 2 (NFATc2), thereby promoting its nuclear translocation [75]. Once in the nucleus, NFATc2 regulates the expression of pro-inflammatory cytokines such as TNF-α and IL-6, consequently creating a neurotoxic inflammatory environment [76]. LRRK2 has been shown to promote the activation of the NLR family CARD domain-containing protein 4 (NLRC4) inflammasome, particularly during bacterial infections like *Salmonella* Typhimurium [77]. This activation involves the phosphorylation of NLRC4 by LRRK2, which enhances the inflammasome’s ability to activate caspase-1 and produce pro-inflammatory cytokines such as IL-1β. Furthermore, studies have indicated that LRRK2 mutations, such as G2019S, can influence the JAK/STAT signaling pathway. This mutation has been demonstrated to suppress the differentiation of regulatory T cells and Th9 cells by altering signal transducer and activator of transcription 3 (STAT3) activity [78]. LRRK2 has been observed to modulate STAT3 phosphorylation, a critical step in its activation. This regulatory impact extends to the expression of cytokines and transcription factors that play a pivotal role in immune responses. Collectively, these studies demonstrate that increased LRRK2 kinase activity enhances the production of pro-inflammatory cytokines, thereby promoting chronic neuroinflammation. The role of LRRK2 in cytokine production underscores its significance in the regulation of neuroinflammatory responses (Figure 5).

### 5.3. Interplay Between LRRK2 and α-Synuclein

The interaction between TLRs and LRRK2 in microglial activation represents a fascinating and critical aspect of neuroinflammatory mechanisms, particularly in neurodegenerative diseases such as Parkinson’s disease [79,80,81]. Toll-like receptors (TLRs) are key pattern recognition receptors on microglia that are tasked with identifying pathogen-associated molecular patterns and damage-associated molecular patterns [82]. Among these receptors, TLR2 and TLR4 are most prominently associated with neurodegeneration. They recognize specific ligands, such as misfolded or aggregated α-synuclein [83,84,85,86]. The binding of α-synuclein to TLRs initiates a signaling cascade within microglia that leads to the upregulation and activation of LRRK2 [75,87]. Upon activation, LRRK2 increases its kinase activity, which drives the production of pro-inflammatory cytokines, exacerbating neuronal damage. Furthermore, increased LRRK2 kinase activity has been demonstrated to promote the generation of nitric oxide via the upregulation of inducible nitric oxide synthase (iNOS), thereby exacerbating oxidative stress and neuronal injury (Figure 5) [88]. This creates a vicious cycle in which the release of neurotoxic substances damages neurons, leading to the release of more α-synuclein, which in turn reactivates TLRs and LRRK2, perpetuating the inflammatory response. Over time, this chronic neuroinflammation accelerates the progression of neurodegenerative disease.

LRRK2 affects pro-inflammatory production that may aggravate neuronal damage. The activation of TLRs by LRRK2 promotes a cycle of neuroinflammation that accelerates neurodegenerative diseases. Thus, chronic neuroinflammation is linked to ongoing neuronal injury and disease progression.

## 6. The Impact of LRRK2 on Neurotrophic Factors in Astrocytes

### 6.1. Neurotrophic Factors and Their Role in Neuronal Health

Astrocytes are essential for CNS homeostasis and neuronal support. They release various neurotrophic factors, which are critical for neuron survival, growth, and differentiation [89]. Astrocytic neurotrophic factor conditioning has been demonstrated to be crucial for the maintenance of neuronal health with aging and responsible for the corresponding neurodegeneration. Thus, the findings from earlier studies reporting neurotrophic factors as being either causes or consequences of PD progression suggest that alterations in the neurotrophic factors’ conditioning by astrocytes are significant. Nerve growth factor (NGF) is crucial for the survival and maintenance of specific neuron populations, including cholinergic neurons. It supports neuronal growth and differentiation and protects neurons from apoptosis [90]. In the context of PD, NGF is particularly important for the maintenance of dopaminergic neurons in the substantia nigra [91]. Brain-Derived Neurotrophic Factor (BDNF) plays a significant role in supporting the survival, growth, and differentiation of neurons in the CNS. It is involved in synaptic plasticity, which is essential for learning and memory [92]. Reduced levels of BDNF have been observed in neurodegenerative diseases, including PD and Alzheimer’s disease (AD) [93,94]. Glial-Cell-Line-Derived Neurotrophic Factor (GDNF) is a potent neurotrophic factor that promotes the survival of various neuronal populations [95,96]. It has been studied extensively for its potential therapeutic effects in PD, as it supports the survival and function of nigrostriatal dopaminergic neurons [97].

### 6.2. Impact of LRRK2 on Neurotrophic Factors in Astrocytes

**Figure 6:**
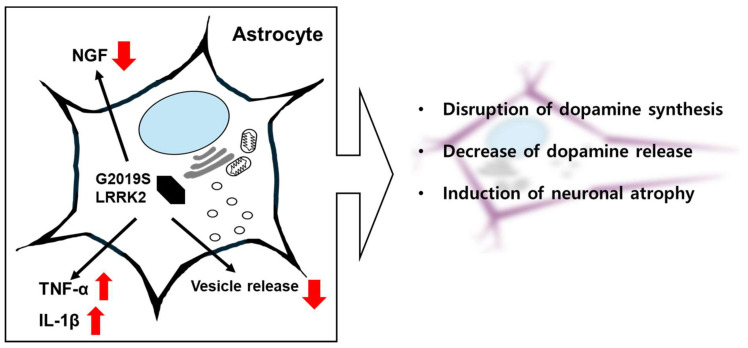
The effects of G2019S LRRK2 expression in astrocytes. G2019S LRRK2 expression has been demonstrated to reduce the release of nerve growth factor (NGF) and increase the secretion of TNF-α and interleukin-1beta (IL-1β) in astrocytes. The vesicle release process, which plays a crucial role in the communication between astrocytes and neurons, has been demonstrated to be altered by G2019S LRRK2 expression in astrocytes. Consequently, the failure of astrocytes to adequately maintain neural health by expressing G2019S LRRK2 has the potential to render dopaminergic neurons vulnerable.

Research has shown that the expression of mutant G2019S-LRRK2 in astrocytes leads to a significant decrease in the levels of NGF. This reduction in NGF impairs dopamine synthesis in dopaminergic neurons, thereby decreasing the release of dopamine [98] (Figure 6). The G2019S LRRK2 mutation increases the levels of proinflammatory cytokines such as IL-1β and TNF-α in astrocytes. These cytokines create a neuroinflammatory environment that exacerbates neuronal damage and degeneration (Figure 6). The shift from a neurotrophic phenotype to a proinflammatory phenotype in astrocytes contributes to the progression of neurodegenerative diseases. Astrocytic asthenia has been observed in patient-derived cultures presenting the G2019S LRRK2 mutation, and this has been demonstrated to be associated with aberrant mitochondrial morphology, decreased mitochondrial activity and ATP production, and increased glycolysis and production of reactive oxygen species. This dysfunction impairs the neurotrophic support provided by astrocytes to neurons, contributing to neuronal degeneration [99]. Astrocytes communicate with neurons through various mechanisms, including the release of extracellular vesicles (EVs) [100]. In a human-induced pluripotent-stem-cell-derived model of PD, the G2019S-LRRK2 mutation in astrocytes was found to induce neuron atrophy. The G2019S LRRK2 mutation alters the biogenesis of these EVs, leading to the abnormal accumulation of PD-related proteins within multivesicular bodies [101]. The altered EVs from astrocytes failed to provide full neurotrophic support to dopaminergic neurons, resulting in their degeneration and atrophy (Figure 6).

LRRK2 in astrocytes is linked to the production of NGF, impairing dopamine synthesis and promoting neuroinflammation. This shift harms neuronal support and is linked to neurodegenerative diseases, as seen in altered extracellular vesicles leading to neuron atrophy.

## 7. The Role of LRRK2 in Ciliogenesis and the Impact of Cilia Defects in Neurons

### 7.1. Role of LRRK2 in Ciliogenesis

**Figure 7:**
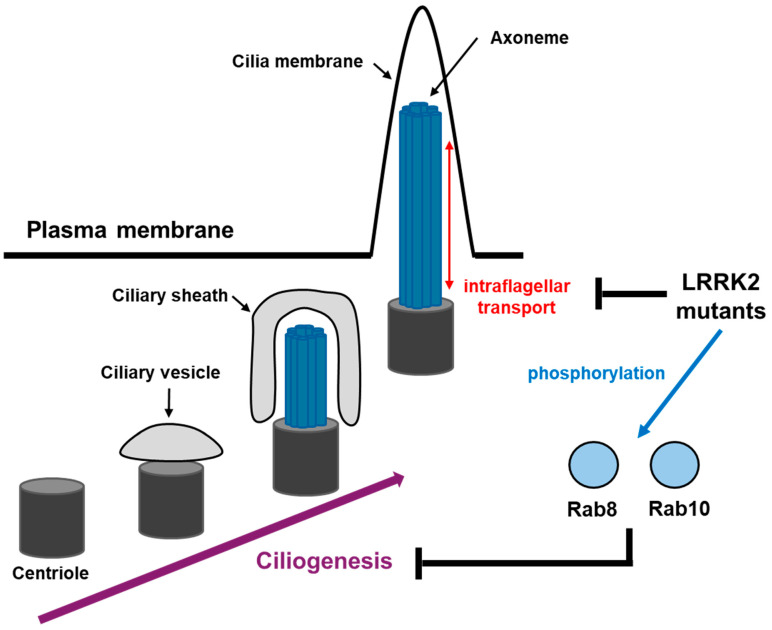
The association of LRRK2 with cilia function and ciliogenesis. The phosphorylation of Rab8 and Rab10 by LRRK2 mutation has been demonstrated to disrupt ciliogenesis in neurons. LRRK2 also has been demonstrated to be associated with intraflagellar transport within the axoneme of cilia. Consequently, aberrant LRRK2 activity has the potential to modify the function of cilia in neural development and signal transduction.

Ciliogenesis, the process whereby cilia are formed, is a fundamental aspect of cellular biology with critical implications for neuronal function [102]. LRRK2 is involved in the stabilization and formation of the primary cilium (Figure 7) [103]. Pathogenic mutations in LRRK2, such as G2019S and R1441C/G, have been shown to impair ciliogenesis [104]. These mutations lead to structural and functional deficits in cilia, disrupting their formation and stability. Notably, LRRK2 regulates the trafficking of ciliary components and proteins, ensuring proper assembly and maintenance of the ciliary structure [105,106]. LRRK2 modulates ciliogenesis through several mechanisms. Firstly, LRRK2 phosphorylates key proteins involved in cilium formation, such as Rab 10 and β-tubulin [107,108]. LRRK2 is also related to the dishevelled family of phosphoproteins and glycogen synthase kinase-3beta (GSK-3β). These interactions influence their activity and localization, which are essential for the proper assembly of the ciliary axoneme, a core structure of cilia composed of microtubules [109]. Thirdly, LRRK2 influences the ciliary transport system, including intraflagellar transport (IFT), which is crucial for the movement of ciliary components along the axoneme [110,111,112]. Disruptions in IFT due to LRRK2 mutations result in defective cilia, impacting their sensory and signaling functions.

### 7.2. Impact of Cilia Defects on Neurons

Cilium defects in neurons have profound implications for neurodevelopment and neurodegeneration. The primary cilia in neurons are involved in critical signaling pathways, such as Sonic Hedgehog (Shh) and Wnt, which regulate neuronal differentiation, axon guidance, and synaptic function [113]. Disruption of these pathways due to defective cilia can lead to various neurological abnormalities. In neurodevelopmental disorders, such as Joubert syndrome and Bardet–Biedl syndrome, cilium defects result in improper neuronal differentiation and migration [114]. This leads to structural brain abnormalities, cognitive deficits, and motor dysfunction [115,116]. In neurodegenerative diseases, including PD and AD, cilium defects have been implicated in the progressive loss of neuronal function [117,118]. Defective cilia disrupt autophagy and vesicle trafficking, leading to the accumulation of toxic protein aggregates [119,120]. Additionally, impaired cilium function affects the maintenance of synaptic connections, contributing to the progressive neurodegeneration observed in these conditions [121,122].

LRRK2 in astrocytes has been linked to the production of NGF, which in turn has been shown to impair dopamine synthesis and promote neuroinflammation. This shift has been demonstrated to have harmful effects on neuronal health and is associated with neurodegenerative disease, as evidenced by altered extracellular vesicles leading to neuronal atrophy.

## 8. Conclusions

LRRK2 is a key regulator of cellular functions, impacting mitochondrial health, mRNA translation, autophagy, neuroinflammation, astrocyte function, and ciliogenesis. Mutations in LRRK2 disrupt these processes, playing a significant role in PD pathogenesis. The convergence of LRRK2’s roles in PD pathogenesis highlights its importance as a therapeutic target. Diverse interventions, ranging from kinase inhibitors and mRNA modulation to mitochondrial restoration and astrocytic support, offer hope for effective treatments. Advances in gene therapy and precision medicine further enhance the prospects for targeted therapies addressing LRRK2-associated PD.

Several LRRK2 inhibitors have been developed and tested in preclinical and clinical studies, targeting the kinase activity of LRRK2 to reduce its phosphorylation of downstream targets [123]. Ongoing research focuses on developing selective and potent LRRK2 inhibitors to maximize therapeutic efficacy while minimizing off-target effects.

While targeting LRRK2 holds therapeutic promise for restoring cellular homeostasis, reducing stress, and delaying senescence, significant challenges remain in translating these findings into effective therapies. The complexity of LRRK2 signaling and its interactions with other pathways require a deeper understanding of its biology. Furthermore, potential off-target effects and the need for long-term treatment pose challenges for developing safe and effective LRRK2-targeted therapies. The development of LRRK2-targeted therapies for PD is challenging due to safety concerns, as LRRK2 inhibitors might disrupt the normal function of LRRK2. To address this issue, the application of diagnostic tools for LRRK2 kinase activity is recommended. Since clinical trials must account for diverse patient populations to gauge effectiveness, genetic variability across global patients further complicates designing universal therapies. Thus, further studies need to bridge the gap between preclinical studies and clinical applications via elucidating the normal function of LRRK2 in various cells and organs and the development of diagnostic and application technologies for dosing criteria for LRRK2 inhibitors.

## Data Availability

The data that support the findings of this study are available from the corresponding author, [I.S.], upon reasonable request due to legal and ethical reason.

## Abbreviations

The following abbreviations are used in this manuscript:

PD	Parkinson’s disease
LRRK2	Leucine-rich repeat kinase 2
ER	Endoplasmic reticulum
Drp1	Dynamin-related protein 1
Mfn	Mitofusin
OPA1	Optic atrophy 1
Ca^2+^	Calcium ion
ROS	Reactive oxygen species
Nrf2	Nuclear factor-like 2
PINK1	PTEN-induced kinase 1
eIF4E	Eukaryotic initiation factor 4E
5′ UTR	5′ Untranslated region
LRS	Leucyl-tRNA synthetase
mTORC1	Mammalian target of rapamycin complex 1
SA-β-gal	Senescence-associated beta-galactosidase
TNF-α	Tumor necrosis factor-alpha
IL-1β	Interleukin-1beta
MAPK	p38 mitogen-activated protein kinase
NF-κB	nuclear factor kappa-light-chain-enhancer of activated B cells
NFATc2	nuclear factor of activated T-cells, cytoplasmic 2
NLRC4	NLR family CARD domain-containing protein 4
TLRs	Toll-like receptors
iNOS	Inducible nitric oxide synthase
NO	Nitric oxide
CNS	Central nerve system
NGF	Nerve growth factor
BDNF	Brain-Derived Neurotrophic Factor
GDNF	Glial-Cell-Line-Derived Neurotrophic Factor
EVs	Extracellular vesicles
GSK-3β	Glycogen synthase kinase-3beta
IFT	Intraflagellar transport
Shh	Sonic Hedgehog (Shh)
